# Epidemiologic, Clinical and Immunological Consequences of Co-Infections during Canine Leishmaniosis

**DOI:** 10.3390/ani11113206

**Published:** 2021-11-10

**Authors:** Erin A. Beasley, Danielle Pessôa-Pereira, Breanna M. Scorza, Christine A. Petersen

**Affiliations:** 1 Department of Epidemiology, College of Public Health, University of Iowa, Iowa City, IA 52242, USA; erin-beasley@uiowa.edu (E.A.B.); dpessoapereira@uiowa.edu (D.P.-P.); breanna.scorza@gmail.com (B.M.S.); 2 Center for Emerging Infectious Diseases, University of Iowa, Iowa City, IA 52242, USA

**Keywords:** co-infections, canine leishmaniosis, risk factors, pathogenesis, immunity, epidemiology

## Abstract

**Simple Summary:**

Canine leishmaniosis (CanL), the most severe, visceralizing form of disease caused by *Leishmania infantum* transmitted by phlebotomine sand flies. CanL is frequently diagnosed in the Mediterranean basin and South America, although it is also found in other regions, including the United States (U.S.). Dogs in these regions are at risk for co-infections, prominently tick-borne diseases. Our review examines epidemiologic, clinical, and immunologic mechanisms found during the most common eight CanL co-infections reported in published literature. Co-infections alter immunologic processes and disease progression impacting CanL diagnosis, therapeutic responses, and prognosis.

**Abstract:**

Canine leishmaniosis (CanL) is a vector-borne, parasitic disease. CanL is endemic in the Mediterranean basin and South America but also found in Northern Africa, Asia, and the U.S. Regions with both competent sand fly vectors and *L. infantum* parasites are also endemic for additional infectious diseases that could cause co-infections in dogs. Growing evidence indicates that co-infections can impact immunologic responses and thus the clinical course of both CanL and the comorbid disease(s). The aim for this review is to summarize epidemiologic, clinical, and immunologic factors contributing to eight primary co-infections reported with CanL: *Ehrlichia* spp., *Anaplasma* spp., *Borrelia* spp., *Babesia* spp., *Trypanosoma cruzi*, *Toxoplasma gondii*, *Dirofilaria immitis*, *Paracoccidioides braziliensis*. Co-infection causes mechanistic differences in immunity which can alter diagnostics, therapeutic management, and prognosis of dogs with CanL. More research is needed to further explore immunomodulation during CanL co-infection(s) and their clinical impact.

## 1. Introduction

Canine leishmaniosis (CanL) is a parasitic disease caused by *Leishmania infantum*, transmitted during phlebotomine sand fly feeding. CanL is endemic across the Mediterranean basin, South America, and parts of Asia and Africa [1,2,3]. Risk of *L. infantum* transmission is associated with changes in competent sand fly distribution, uncontrolled importation of infected dogs to non-endemic areas, and vertical transmission to offspring [4,5,6,7]. CanL presentation can range from subclinical disease, lymphadenopathy, and dermatologic lesions to advanced chronic renal disease [8]. The timeline of CanL progression varies between dogs, and factors contributing to CanL progression are poorly understood. Other vector-borne diseases are primarily found within the regional distribution of *L. infantum* (Table 1). Many reports indicated increased odds of co-infection with other vector-borne and infectious diseases when a dog was diagnosed with CanL [9,10,11,12]. Co-infection with other vector-borne diseases can hasten CanL progression.

In this review, we use the term co-infection to indicate presence of the pathogen’s nucleic material or direct evaluation of both *L. infantum* and another pathogenic microbe. Co-exposure within this review indicates detection of antibodies against one or both pathogenic microbes. We recognize the significance of both co-exposures and co-infections, as confirmed co-infection can be diagnostically challenging. The effects of these co-infections upon the epidemiology, immunologic responses, clinical presentation, and CanL management are important for both clinical and molecular understanding. This review provides an overview of CanL and highlights primary co-infections found in dogs.

## 2. Overview of CanL

The predominance of *L. infantum*-infected dogs develops subclinical infection. However, 5–10% of the infected dogs eventually progress to chronic visceral disease within months to years after infection [49]. The factors contributing to progression from subclinical to clinical disease are under active investigation. However, it has been reported that due to genetic mutations encoding protective responses of macrophages, Ibizan hounds are protected against *Leishmania* infection, and boxers are susceptible to *Leishmania* infection [50,51,52].

Clinical CanL can develop at any time within a dog’s life and may progress years after initial infection. Signs can range from weight loss, mild lymphadenomegaly, papular to nodular lesions or ulcerative dermatitis, epistaxis, thrombocytopenia, and nonregenerative anemia to advanced renal disease and splenomegaly and hepatomegaly [8,53]. In Pereira et al. (2020), the average age of presentation with CanL was 4–8 years old, 59.6% were male, 67.4% of dogs evaluated had dermatologic signs, 60.4% had anemia, 52.8% had hyperproteinemia, and 43.2% had uremia [54]. In Europe, the LeishVet clinical staging guidelines are used to monitor progression of CanL and guide appropriate disease management [8]. By LeishVet stage 2, there is usually evidence of hypergammaglobulinemia and hypoalbuminemia, consistent with non-specific B cell activation seen with many infectious diseases [8]. Signs of renal disease characterize LeishVet stages 3 and 4 [8]. The most advanced stage, stage 4, is distinguished by International Renal Interest Society (IRIS) stages 3–4, manifested by proteinuria, uremia, and nonregenerative anemia [8]. Chronic kidney disease is a major cause of death for dogs with CanL [8].

Polymerase chain reaction (PCR) methods and quantitative serological methods including enzyme-linked immunosorbent assay (ELISA) and immunofluorescent antibody test (IFAT) were more commonly used for diagnosis of CanL than direct evaluation of parasites from culture or cytology/histopathology [8,55]. Blood or sera samples are often used due to ease of collection, although lymph node or splenic aspirates may be more sensitive [8]. Depending on the dog’s clinical status, routine monitoring every 3–6 months is warranted in order to provide appropriate therapy [55]. After reviewing common co-infections and associated immune responses, we provide comments on treatment and prevention of CanL and co-infections.

## 3. Immune Responses during CanL

**Figure 1:**
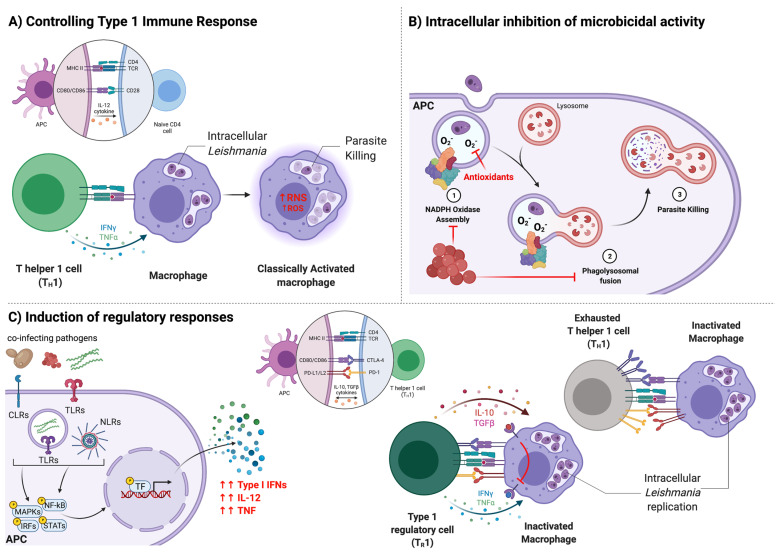
Types of immunological interference with anti-*Leishmania* responses. (**A**) A controlling Type 1 immune response occurs when *Leishmania* antigen presenting cells (APCs) express interleukin-12 (IL-12) to polarize *Leishmania*-specific CD4^+^ T cells to T helper type 1 (Th1) cells. Th1 cells express interferon-gamma (IFN-γ) after encountering a parasitized macrophage, which activates anti-microbicidal pathways including predominantly reactive oxygen species (ROS), and compared to murine models, in dogs less inducible nitric oxide synthase (iNOS)-driven reactive nitrogen species (RNS) production by macrophages and killing of intracellular parasites. (**B**,**C**) Co-infections may utilize these mechanisms that interfere with a controlling Type 1 immune response described in panel (**A**); (**B**) Intracellular pathogens inhibit macrophage microbicidal activity at multiple levels. Inhibition of nicotinamide adenine dinucleotide phosphate (NADPH) oxidase assembly on the phagosomal membrane prevents oxidant generation while production of antioxidants within the phagosome can quench the pathogen damaging effects of ROS. Inhibition of phagolysosomal fusion prevents acidification of the phagosome and release of hydrolytic enzymes contained within the lysosome meant to destroy engulfed pathogens; (**C**) Co-infecting pathogens can trigger inflammatory cytokine production by APCs via Toll-like receptors (TLRs), Nod-like receptors (NLRs), and C-type lectin receptors (CLRs). Inflammation can trigger induction of regulatory pathways, such as expression of inhibitory receptors including programmed cell death protein 1 (PD-1) and CTLA-4 on Th1 cells and inhibitory ligands on myeloid cells. Inflammation triggers regulatory cytokine production (IL-10 or transforming growth factor beta (TGF-β)) by innate and adaptive cells. Regulatory signals cause Th1 cells to differentiate into Type 1 regulatory cells (Tr1) co-expressing IFN-γ and IL-10. IL-10 antagonizes the activating effects of IFN-γ on macrophages thus negating microbicidal activation and parasite outgrowth. If chronic inflammation persists in combination with prolonged T cell receptor (TCR) signaling, *Leishmania*-specific Th1 cells further upregulate inhibitory receptors and can become exhausted. Exhausted Th1 cells no longer produce IFN-γ in response to *Leishmania* antigen, thus macrophages receive no exogenous activation signals, and parasite replication occurs unchecked.

The clinical outcome of CanL is determined by a multitude of factors including host and parasite genetics, husbandry and host immune responses [8]. Immune-mediated mechanisms either allow the *L. infantum* parasites to replicate within host cells or resist parasite replication via innate and adaptive anti-parasitic immune responses (Figure 1) [56,57].

*Leishmania* spp. are obligate intracellular protozoan parasites; therefore, replication and survival are accomplished by infecting host cells. After initial transmission into a canine host, *L. infantum* promastigotes are rapidly taken up by phagocytic cells, primarily neutrophils, monocytes, macrophages, and dendritic cells [58]. *Leishmania* parasites preferentially reside within macrophages, where they differentiate from promastigote to amastigote forms, replicate, and establish a long-lasting intracellular infection [59,60]. Macrophages are highly specialized immune cells for neutralizing and eliminating intracellular pathogens [61] (Figure 1A). During phagocytosis, phagosomes containing *Leishmania* promastigotes usually merge with lysosomes containing hydrolytic enzymes and an acidic environment that kill promastigotes [59,62]. Macrophages also produce reactive oxygen species (ROS) in order to kill intracellular parasites and limit their replication [63,64,65]. Although those innate immune responses typically pose a challenge for invading parasites, *Leishmania* has developed numerous evasion mechanisms to facilitate their long-term survival inside macrophages [66,67,68,69]. Inside the phagolysosome, *L. infantum* amastigotes resist the hostile environment by delaying phagolysosome fusion [70,71] and producing antioxidants to counteract the reactive free radicals [72,73,74]. Ultimately, to prevent *L. infantum* parasite replication and consequent disease progression, macrophages must rely on *Leishmania*-specific adaptive immune responses to effectively overcome *Leishmania* defenses and elicit their killing functions [56,57,75].

Protective immunity against *Leishmania* infection in dogs, as in humans, requires the development of a predominant T helper type 1 (Th1) immunity, characterized by induction of interferon gamma (IFN-γ)-producing CD4^+^ T cells [56,75,76]. Early after initial infection, parasite-host interactions lead to transcription and secretion of pro-inflammatory cytokines [77,78,79]. Interleukin-12 (IL-12) promotes naïve CD4^+^ T cell differentiation into effector Th1 cells, which proliferate and produce IFN-γ, tumor necrosis factor alpha (TNF-α), and IL-2 [80,81,82]. Among these, IFN-γ is crucial for controlling *Leishmania* infection, by activating macrophages through inflammatory cytokine and chemokine production, upregulating antigen presentation machinery within the macrophage, and sustaining microbicidal responses [75,83,84]. Subclinical infection is mainly characterized by the absence of detectable *L. infantum* DNA in peripheral blood via quantitative PCR (qPCR) due to activated macrophages controlling parasite replication and keeping parasite burden low [49]. Subclinical dogs CD4^+^ T cells are able to proliferate and produce IFN-γ after in vitro *Leishmania* antigen stimulation [78,85,86]. IL-10 is a regulatory, anti-inflammatory cytokine that antagonizes IFN-γ-mediated responses, preventing excessive inflammation and dampening microbicidal responses important for parasite clearance [49,87]. Low amounts of IL-10 produced during subclinical infection may serve as negative feedback to limit Th1-induced inflammation without compromising host cell parasite killing abilities [75,87]. A balance between inflammatory and regulatory T cell responses is required for controlling parasite replication over time while minimizing exacerbated inflammation that may cause damage to the host [49]. As *Leishmania* infection is seldom sterilely cured, a constant ongoing Th1 immune response is needed to maintain a subclinical state [49,75].

After months to years without resolving infection, prolonged cellular immune response activation and production of pro-inflammatory cytokines eventually promote increased generation of IFN-γ/IL-10 co-producing Type 1 Regulatory T (Tr1) cells [49,75] (Figure 1C). At high enough levels, IL-10 renders macrophages unresponsive to IFN-γ, thereby inhibiting the enhanced microbicidal responses and contributing to parasite survival [49,75]. As *L. infantum* parasites continue to propagate, long-term exposure to *Leishmania* antigen can lead to T cell exhaustion—defined by progressive T cell hypo-responsiveness and significant increased expression of inhibitory receptors on CD4^+^ T cells such as programmed cell death protein 1 (PD-1), LAG3, and CTL-4 [76,88,89]. During disease progression, *Leishmania* parasites benefit from the immunosuppressive properties of IL-10 and dysfunctional CD4^+^ T cells. Thus, clinical CanL is characterized by decreased *Leishmania*-specific CD4^+^ T cell proliferation and production of IFN-γ, increased IL-10 production and parasite loads in different tissues, and high antibody levels detected by ELISA [49].

It has been recognized that *Leishmania* parasites occur concomitantly with other pathogens in infected dogs [12]. Altered or defective immune responses promoted by subsequent co-infections may facilitate CanL progression. During their lifetime, *L. infantum*-infected dogs may be independently exposed to various bacterial, parasitic, fungal, and viral infections. These co-infections can result in synergistic interactions that may consequently impact *L. infantum* infection diagnosis, disease severity, and treatment [10,49]. Therefore, understanding the interactions between *Leishmania* parasites and other relevant pathogens could help the development of better prevention, diagnosis, and treatment strategies.

## 4. Bacterial Co-Infections

### 4.1. Ehrlichia spp.

#### 4.1.1. Microbe and Epidemiology

*Ehrlichia* spp. are obligate intracellular Gram-negative bacteria from the family *Anaplasmataceae,* order Rickettsiales. These tick-borne bacteria are commonly found in the Southern U.S., Brazil, and Mediterranean basin—where *L. infantum* is endemic among dogs [10,18,90]. Dogs are most frequently infected by *E. canis*, the etiologic agent of canine monocytic ehrlichiosis [91,92,93].However, it has been reported that *E. ewingii* and *E. chaffeensis* also naturally infect canids [13]. *E. canis* is primarily transmitted by *Rhipicephalus sanguineus* ticks, and *E. ewingii* and *E. chaffeensis* are most frequently transmitted by *Amblyomma americanum* ticks [13,14,15].

Recent epidemiological studies have found a strong association between *Ehrlichia* spp. co-infections and CanL progression [9,10]. *L. infantum* and *E. canis* co-occurrence is one of the most common co-infections of dogs [10,12,90,94]. In Brazil, 31.75% of 200 dogs with CanL, as diagnosed by Dual-Path Platform (DPP)^®^ Canine Visceral Leishmaniosis serological test and ELISA, were co-exposed with *Ehrlichia* [95]. Furthermore, Toepp et al. (2019) found 41.67% of dogs in Northeastern Brazil with clinical leishmaniosis were co-exposed to *Ehrlichia* spp. [10]. In Spain, 56% (34/61) of dogs with clinical CanL were co-infected with *Ehrlichia* [12]. *Ehrlichia* co-infections have been reported in Nepal and co-exposures in Germany at lower incidences than Brazil and Spain [96,97]. Dogs with CanL were more likely to be *E. canis*-seropositive than clinically healthy dogs from the same endemic area [9,10]. For instance, dogs infected with *E. canis* had 12.4 times the odds of clinical CanL than control dogs (*p* = 0.022) in Cyprus [9].

#### 4.1.2. Clinical Disease and Biochemical Findings

Although dogs may be subclinical during *Ehrlichia* infection, it can cause a wide range of clinical signs. Signs can be non-specific and include fever, lethargy, cachexia, pale mucous membranes, petechiae, ecchymoses, epistaxis and gross lesions such as lymphadenomegaly, splenomegaly, and hepatomegaly [92,98]. These signs overlap with those presented during CanL. If untreated, canine monocytic ehrlichiosis can be fatal [92]. *Ehrlichia* infection is usually diagnosed by screening for *Ehrlichia*-specific antibodies via ELISA or IFAT [92]. *Ehrlichia* infection can also be identified through microscopic evaluation of blood smears, where inclusion bodies are visible; however, direct evaluation of morulae has low sensitivity [92]. Cardinot et al. (2016) evaluated brain tissue of dogs with known CanL and found 58.3% (of 24 dogs) were co-infected with *L. infantum* and *E. canis*, and 83.3% were infected with *E. canis* overall [99].

Noteworthy, both infections may promote similar biochemical and hematological abnormalities [11,100], which supports a synergistic effect between these two pathogens in promoting disease severity. In Brazil, Andrade et al. (2014) reported chronic inflammatory reactions in lymphoid tissues, increased total plasma protein and globulin concentrations, thrombocytopenia, and normocytic normochromic anemia in dogs solely infected with *L. infantum* and those co-infected with *E. canis* [101]. However, co-infected dogs presented with twice as many dermal amastigotes compared to dogs infected with *L. infantum* alone [101]. These dogs also had significantly decreased albumin concentrations, and more evident anemia, characterized by lower erythrocyte count, hemoglobulin levels, and hematocrit [101]. Similar findings were found by Baxarias et al. (2018) in Spain, where *E. canis*-seropositive dogs presented with increased total protein and gamma globulin levels, and decreased albumin concentrations, decreased red blood cells, hemoglobulin levels, and hematocrit [12]. Regarding hemostatic parameters, co-infection of *L. infantum* and *E. canis* decreased platelet aggregation responses and increased activated partial thromboplastin time (aPTT) [50,102].

#### 4.1.3. Immunological Effects

*Ehrlichia* spp. may contribute to CanL progression through diverse immunological mechanisms. Similar to *Leishmania* parasites, monocytes and macrophages are natural host cells for *Ehrlichia* spp. [103]. *Ehrlichia* can infect macrophages within tissues already affected by *L. infantum* infection, such as lymph nodes, spleen, liver, and bone marrow. Inside the host cell, *Ehrlichia* spp. replicate within dense membrane-bound vacuoles in the cytoplasm [104].

To avoid destruction by host cells, *Ehrlichia* spp. have developed several immune evasion mechanisms to ensure survival and replication. Unlike most Gram-negative bacteria, *Ehrlichia* spp. do not synthesize lipopolysaccharide (LPS) or peptidoglycan (PG) [105,106], structural components of the bacterial cell wall. Both LPS and PG are pathogen-associated molecular patterns (PAMPs) able to induce innate immune signaling pathways by binding to pattern recognition receptors (PRRs) expressed by host cells [107,108,109]. Furthermore, like *Leishmania* parasites, *Ehrlichia* spp. can inhibit lysosomal fusion to their vacuoles and prevent destruction by host proteases, esterases, and acidification (Figure 1B) [110,111]. *E. canis* impaired antigen-presentation by DH82 cells (a canine macrophage cell line) by downregulating surface expression of major histocompatibility complex (MHC) II [112]. Therefore, *Ehrlichia* spp. internalization may inhibit strong innate immune responses—favoring *L. infantum* survival within co-infected host cells.

Different pathways for *Ehrlichia* infection and survival in the host may present beneficial molecular environments for co-infection with *Leishmania*. *E. chaffeensis*-infected monocytes become less responsive to external stimuli (such as *Escherichia coli*-derived LPS), which decreased activation of p38 mitogen-activated protein kinase (MAPK) and extracellular signal-regulated protein kinases 1 and 2 (ERK1/2) in host cells [113]. This resulted in decreased signaling downstream of Toll-like receptors 2 and 4 (TLR2/4) and CD14 [113]. Agallou et al. (2014) demonstrated that *L. infantum* internalization by peritoneal macrophages impairs activation of p38 MAPK and ERK1, which downregulated expression of transcription factors and their target genes required for promoting microbicidal responses and cytokine production [114]. Through microarray analyses, Zhang et al. (2004) reported that *E. chaffeensis* infection in THP-1 cells (a human monocyte cell line) suppressed transcription of pro-inflammatory cytokines involved in stimulating Th1-mediated immunity, such as IL-12/18 [115]. Therefore, *Ehrlichia* spp. and *L. infantum* may synergistically inhibit MAPK signaling pathways and the induction of Th1-mediated responses, thus impairing macrophage effector functions. In a mouse model of fatal ehrlichiosis, *Ehrlichia* induced strong pro-inflammatory responses via activation of inflammasomes, which promoted production of IL-1β and Type I IFNs [116]. Some mouse model studies demonstrated that production of Type I IFNs, IFN-α and IFN-β, led to impaired Th1 cell responses during visceral leishmaniasis [117,118]. Type I IFN production by *Ehrlichia* co-infected macrophages may impact immune responses against *L. infantum* in infected dogs and then prompt disease progression.

*Ehrlichia* spp. lack most enzymatic ROS-scavenging mechanisms required for ROS detoxification [119]. *Ehrlichia* inhibit or block superoxide generation by human macrophages via degradation of nicotinamide adenine dinucleotide phosphate (NADPH) subunit p22^phox^, preventing NADPH assembly on the phagosomal membrane (Figure 1B) [120]. In addition, *Ehrlichia* induces host mitochondrial upregulation of manganese SOD (MnSOD) in THP-1 cells, preventing ROS-induced apoptosis and contributing to its intracellular survival [121]. Noteworthy, *E. ewingii* was also shown to delay apoptosis in infected canine neutrophils in vivo via stabilization of mitochondrial membrane permeability [122]. Liu et al. (2011) reported that *E. chaffeensis* can inhibit mitochondrial metabolism in infected DH82 cells, preventing host cell apoptosis, but the underlying mechanism was not known [123]. By blocking superoxide generation and preventing apoptosis, *Ehrlichia* spp. could facilitate *L. infantum* infection and prolong the life of co-infected cells.

Similar to immune responses against *Leishmania* parasites, production of IFN-γ by CD4^+^ Th1 cells is crucial for conferring protection against *Ehrlichia* infection [124]. *Ehrlichia* infection in human monocytes readily resolve infection in vitro if pre-treated with exogenous IFN-γ, but no resolving effect was observed if treatment was made after the establishment of infection [125]. This implies that intracellular *Ehrlichia* renders macrophages less responsive to IFN-γ. Lee et al. (1998) reported that *E. chaffeensis* impaired JAK-STAT signaling in peripheral blood mononuclear cell (PBMC)-derived monocytes and THP-1 cells early after IFN-γ treatment, which was thought to be partially mediated by upregulation of protein kinase A (PKA) activity [126]. JAK-STAT signaling mediates several biological processes, including induction of microbicidal responses in infected cells. *Leishmania* parasites also inhibit IFN-γ-induced JAK-STAT signaling in macrophages [127]. In this context, *Ehrlichia* spp. and *Leishmania* parasites may synergistically act to inhibit JAK-STAT signaling in co-infected canine host cells, contributing to *Leishmania* survival within those cells by interfering with IFN-γ pathway elements (Figure 1C).

### 4.2. Anaplasma spp.

#### 4.2.1. Microbe and Epidemiology

*Anaplasma* spp. are obligate intracellular Gram-negative bacteria from the Rickettsiales order. *A. phagocytophilum* are transmitted by bites of *Ixodes scapularis* and *Ixodes pacificus* ticks in North America and *Ixodes ricinus* in Europe [18,19]. *A. platys* is believed to be vectored by *Rhipicephalus sanguineus* ticks in Brazil and Europe [9,16,20]. *A. phagocytophilum* and *A. platys* infect dogs [18]. Surveillance in dogs is usually performed via serology, which does not always differentiate *A. phagocytophilum* and *A. platys* exposure [18]. *A. platys* is the predominant species infecting dogs in Brazil [16] and can be zoonotic. However, due to its increased ability to cause human disease, there is more literature available regarding pathogenesis of *A. phagocytophilum*.

*Anaplasma* spp. are endemic in *L. infantum*-endemic regions including Europe, South America, and the United States [128]. Toepp et al. (2019), found that approximately 33% of dogs were exposed to *Anaplasma* in Natal, Brazil [10]. In Spain, dogs with clinical leishmaniosis were significantly more likely to be exposed to *A. phagocytophilum* than presumed healthy dogs (OR = 14.3, *p* = 0.002) [12]. *A. phagocytophilum* exposure was associated with increased serum total protein, gamma globulin, and decreased serum albumin in dogs with CanL [12]. In another study from Spain, co-infection with *Anaplasma* spp. significantly increased the risk of leishmaniasis by 79% [90]. Additionally, 3.8% of 507 dogs with signs for a vector-borne disease were seropositive for *Anaplasma* spp. compared to 2.1% of 556 dogs without signs in a study by Miró et al. (2013) [129]. Among dogs with clinical CanL in Cyprus, 4% had DNA sequences for *A. platys*, compared to 3% of dogs without CanL, and 10% of dogs were serologically positive for *A. phagocytophilum/platys* compared to 2% of dogs without CanL, although not statistically significant [9,20]. From a Brazilian study, 18% of 66 dogs with CanL were co-exposed with *Anaplasma* spp. [130].

#### 4.2.2. Clinical Disease and Hematologic Findings

Dogs infected with *Anaplasma* spp. can have signs like ehrlichiosis, and in turn, like CanL. Hematologic changes can include increased gamma globulin levels and decreased albumin, anemia, and thrombocytopenia [12].

#### 4.2.3. Immunological Effects

*A. phagocytophilum* infects granulocytes, while *A. platys* infects thrombocytes and monocytes systemically, resulting in anaplasmosis [128]. This can lead to acute febrile illness with anemia, although subclinical infection occurs frequently [131]. If not identified and treated, infection can persist for several months post-infection [132]. Due to its propensity to infect myeloid cells, the bone marrow is a major site of infection and increased inflammatory cytokines were measured in bone marrow cells from experimentally infected mice [133]. Immune responses to CanL may be affected by cytopenias caused by anaplasmosis including lymphocytopenia, neutropenia, and thrombocytopenia [134]. Like leishmaniosis, nonregenerative anemia is a hallmark of anaplasmosis and thought to be a consequence of bone marrow infection and alterations of precursor populations [133]. *Anaplasma* infection may synergistically induce anemia in dogs with CanL [134].

As observed with *Leishmania*, *Anaplasma* have evolved several immune evasion strategies to replicate intracellularly in myeloid host cells. *Anaplasma* bacteria have also lost the major PAMPs PG and LPS, which allow a more silent entry into immune cells [131]. Indeed, neither NFκB nor p38 MAPK activation are observed following in vitro infection of monocytes with *A. phagocytophilum* [135]. *Anaplasma* infection has been shown to reduce key neutrophil functions associated with killing intracellular pathogens such as decreased expression of proteins critical to formation of the NAPDH oxidase leading to decreased oxidative burst (Figure 1B) [131,136]. In addition, *A. phagocytophilum* prevents lysosome fusion with the phagosome in infected neutrophils [137].

After an inflammatory initial acute phase, a refractory period emerges with decreased immune function in some cases of anaplasmosis [137,138,139]. *Anaplasma* may impact the anti-*Leishmania* immune response by exacerbating immunosuppression. Neutropenia, combined with neutrophil dysfunction, can leave the host susceptible to pyogenic opportunistic infections such as *Staphylococcus aureus* and *Listeria monocytogenes* [18] and certainly also *Leishmania*. This proclivity to intracellular pathogen infection after anaplasmosis indicates broad suppression of intracellular pathogen control mechanisms which may be shared by cells attempting to control intracellular *Leishmania* replication.

Lymphocytopenia experienced during CanL may be compounded with further lymphocytopenia caused by *Anaplasma* [134]. Decreased peripheral CD4^+^ T cells are observed, and Th1 responses are crucial for controlling *L. infantum* burden in infected dogs. In sheep infected with *A. phagocytophilum*, the frequency of IL-2Rα expressing CD4^+^ T cells was transiently significantly decreased in circulation [138]. The decreased ability to sense IL-2 may be related to the transient decrease in lymphocyte proliferation to tetanus toxoid and mitogen and decreased IFN-γ production by whole blood cells in response to mycobacterial antigen also observed [138]. Further, IFN-γ signaling is disrupted in *Anaplasma* infected cells by decreased expression of IFNGR1 and altered STAT1 activation (Figure 1C) [131]. Serum IL-10 was measured in animals with anaplasmosis which may promote off-target regulatory responses in *Leishmania*-infected cells or *Leishmania*-specific effector cells [131,140]. CD4^+^ T cell ability to proliferate and express IFN-γ are associated with control of leishmaniosis. Immune changes from *Anaplasma* infection promote detrimental effects on CD4^+^ T cells and may lead to *Leishmania* proliferation and enhanced leishmaniosis.

### 4.3. Borrelia spp.

#### 4.3.1. Microbe and Epidemiology

*Borrelia burgdorferi*, causative agent of Lyme disease, is a bacterial spirochete transmitted by *Ixodes scapularis* and *Ixodes pacificus* in North America [21,22,23]. Dogs are incidental hosts for *Bo. burgdorferi* and not part of the transmission cycle [141]. In North America, canine Lyme disease has been significantly associated with *Bo. burgdorferi* sensu stricto (ss) strains, whereas in Europe, most cases are associated with sensu lato (sl) species (*Bo. garinii* and *Bo. afzelii*) [22,23].

*Bo. burgdorferi* and *L. infantum* co-infections among dogs living in endemic areas have been reported in recent epidemiological studies [10]. In a study with dogs from the U.S., 33.33% of the dogs with clinical CanL had exposure to *Bo. burgdorferi* [10]. Little et al. reported that *Bo. burgdorferi* infection in dogs remains widespread in the U.S., where specific antibodies were detected in 5.9% of the dogs tested via in-clinic ELISA (SNAP^®^ 4Dx^®^ Plus Test) [142]. The prevalence rate was higher in the Northeastern U.S. (12.1%), where *L. infantum* is also enzootic among dogs [142]. Although the U.S., particularly the Eastern U.S., has relatively high seropositivity for canine borreliosis [143], there is currently limited published data on co-infection with CanL. In Europe, canine *Borrelia* spp. infections have been reported to be low, with no dogs being seropositive in a Cyprus study [20] and only 0.4% (of 1100 dogs) of dogs being seropositive in a Spanish study [129]. Dogs in Europe have been infected with *Bo. garinii* or *Bo. afzelii*, transmitted by *Ixodes ricinus* [19,22,24]. As dogs in Europe are at risk of exposure to *Borrelia* spp. and *Leishmania*, it is possible to have co-infections in that region; however, we were not able to find any reports specifically documenting this co-infection.

#### 4.3.2. Clinical Disease

Dogs seropositive for *Bo. burgdorferi* are largely subclinical (up to 95%) [144]. When clinical signs do occur, arthritis, lameness, lymphadenopathy, anorexia, weight loss, and fever are most commonly observed [144]. In advanced clinical cases, Lyme nephritis is possible, and signs and laboratory findings consistent with acute or chronic kidney disease can be exhibited [144]. Diagnosis of canine borreliosis may require a combination of tests, depending on presentation of clinical signs, and can include rapid serological tests, quantitative C6 protein ELISA, and immunofluorescent assays (IFAs) [145].

#### 4.3.3. Immunological Effects

*Bo. burgdorferi* does not produce LPS [146]; however, lipoproteins found in its outer membrane can activate pathogen recognition receptors and initiate pro-inflammatory signaling. *Bo. burgdorferi* ligands can be recognized by TLR1/2, TLR7/8, and TLR9 and activate production of inflammatory cytokines, such as TNF-α, IL-6, IL-12 and pro-IL-1β [147,148]. These cytokines induce polarization of a mixed Th1 and Th17 cell response, leading to production of IFN-γ and IL-17, which is highly inflammatory [149]. The Nod-like receptor (NLR) NOD2, interacting with RIP2, was also shown to recognize borrelial compounds in mice leading to IL-1β, IL-6, TNF-α, IL-8 and IL-10 production [150]. NOD2 is an intracellular PG sensor [150]. Jutras et al. (2019) demonstrated that *Bo. burgdorferi* release muropeptides (PG fragments) into the extracellular environment instead of recycling them for remodeling their PG cell wall [151]. Both PG and antibodies against *Bo. burgdorferi* PG were shown to be detectable in synovial fluids from Lyme arthritis human patients before and after treatment, suggesting that muropeptides may persist long after *Bo. burgdorferi* active infection [151]. Indeed, these molecules elicit persistent inflammatory responses in stimulated human PBMCs and cause severe inflammation in mouse joints [151].

TLRs and NLRs engagement by *Bo. burgdorferi* can also induce production of Type I IFNs in isolated human monocytes and mouse in vivo models [152,153,154,155]. The role of Type I IFN on host defense to non-viral pathogens is complex and can lead to different outcomes. However, Type I IFN signaling is likely to be modulated during visceral leishmaniasis. In an infection model, *L. infantum* induced Type I IFN expression in conventional dendritic cells in vivo, which lead to an impaired Th1 cell response [118]. Recently, Kumar et al. found high levels of IFN-α, IFN-β, and their receptors in PBMCs from visceral leishmaniasis patients before drug treatment relative to post-treated VL patients and endemic controls [117]. In vitro studies with human PBMCs and in vivo mouse models demonstrated that Type I IFN signaling can suppress *Leishmania*-specific IFN-γ production by effector CD4^+^ Th1 cells [117], which was found to contribute to disease progression. It has been demonstrated in mice that chronic viral infections with Type 1 IFN signaling can alter the immune cell composition within the spleen and lead to immunosuppressive states [156,157,158]. Dogs with CanL also undergo progressive breakdown of splenic architecture, which may be compounded by Type I IFNs induced by *Borrelia* [159].

*Bo. afzelii* and *Bo. garinii* infections seem to be less aggressive than *Bo. burgdorferi* infection [160,161]. After stimulating monocyte-derived macrophages from healthy human donors with different *Bo. burgdorferi* isolates, Strle et al. (2009) found that U.S. *Bo. burgdorferi* isolates induced significantly higher IL-6, IL-8, CCL3, CCL4, and TNF-α secretion compared with European *Bo. afzelii* or *Bo. garinii* isolates [162]. Consistently, production of IL-6, CCL3, CCL4, and TNF-α were found to be significantly higher in serum of *Bo. burgdorferi*-infected patients than in *Bo. afzelii*- or *Bo. garinii*-infected patients or healthy controls [162]. One study demonstrated that *Bo. afzelii* spirochetes induce significantly more IL-17A production by Lyme disease patients’ PBMCs compared to that induced by *Bo. burgdorferi* sensu stricto spirochetes [163]. However, Lyme disease patients’ PBMC-derived IL-17A, IL-17F, and IL-22 proteins were induced by all three *Borrelia* species compared to unstimulated PBMCs, which highlights that *Borrelia* spp. induce this inflammatory pathway [163].

Conflicting reports have suggested that Th17-mediated immune responses might have protective and/or pathological roles during visceral leishmaniasis. In an experimental model of CanL, Hosein et al. (2015) found progressive downregulation of Th17-related cytokine gene expression in lymph nodes and spleen, which was associated with a silent, asymptomatic establishment of *L. infantum* infection [164]. However, exogenous IL-17A synergizes with IFN-γ in a dose-dependent manner to increase nitrite levels and reduce intracellular parasite burden in murine macrophages [165]. Other studies have shown that IL-17 production can lead to recruitment of high numbers of neutrophils and macrophages to inflammatory sites, which may lead to tissue destruction observed during cutaneous leishmaniasis [166,167]. *L. infantum* co-infections with *Bo. burgdorferi* sl species inducing Th17 immune responses in dogs may contribute to immunopathology.

While an inflammatory response is thought to contribute to *Bo. burgdorferi* infection control, sustained inflammation in the presence of chronic T cell receptor engagement can result in upregulation of inhibitory receptors such as PD-1, TIM-3 and CTLA-4 on T cells [168]. Prolonged upregulation of multiple inhibitory receptors has been shown to lead to exhaustion [168]. Therefore, during *Bo. burgdorferi* and *L. infantum* co-infection in dogs, chronic inflammation might contribute to T cell exhaustion, leading to *Leishmania* uncontrolled replication and CanL progression (Figure 1C).

## 5. Protozoal Co-Infections

### 5.1. Babesia spp.

#### 5.1.1. Microbe and Epidemiology

*Babesia* are tick-borne protozoan Piroplasmida parasites. The sporozoite life stage inoculated by the tick invades host erythrocytes, where they differentiate and replicate until the erythrocyte ruptures, and merozoites invade new erythrocytes spreading the parasite throughout the bloodstream [25]. Diagnosis is made based on visualization of parasite forms on blood smear or IFA, while PCR or reverse line blot is required for speciation [169]. Several species of *Babesia* are known to infect dogs, including *Ba. canis* (transmitted by *Dermacentor reticulatus* ticks)*, Ba. vogeli* (transmitted by *Rhipicephalus sanguineus* ticks)*, Ba. gibsoni* (transmitted by *Haemophysalis* spp. ticks)*,* and *Ba. microti*-like isolates in Europe [25,26,28,29,86].

Due to its high seroprevalence among dogs in South America, Europe, and the U.S., several studies have documented co-infection with *Babesia* spp. in *L. infantum* infected dogs. In the U.S., *Babesia* exposure was approximately 32% among a cohort of dogs living with *L. infantum* [10]. In Northern Portugal, *L. infantum* was the most prevalent co-infecting agent among a cohort of dogs with babesiosis using PCR to detect *L. infantum* [170]. Cardoso et al. (2010) note that studies not using PCR to detect *L. infantum* underestimated rates of co-infections [170]. This study found that the *L. infantum* and *Ba. canis* co-infected dogs did not experience lower hematocrit values compared to *Ba. canis* singly infected dogs; however, a complete clinicopathological evaluation was not performed, and a higher proportion of the co-infected dogs (22%) succumbed to disease compared to singly infected dogs (6%) [170]. In Brazil, *Babesia*-seropositive dogs can be found in every state, with some areas reporting as high as 67% canine seropositivity [28]. The true rate of co-infection between *L. infantum* and *Babesia* species is also complicated by the low sensitivity of *Babesia* PCR from peripheral blood in non-clinical dogs. In a study from Brazil, 81.6% of a canine cohort was seropositive for *Babesia* exposure, with 25% co-exposed with *L. infantum*, but only 3.3% were PCR-positive for *Babesia* [171]. Whether the large seroprevalence of *Babesia* in dogs from endemic areas are currently subclinically infected or have resolved infection is not clear.

#### 5.1.2. Clinical Disease and Biochemical Findings

Different *Babesia* species are known to elicit different clinical manifestations and pathogenesis in canine hosts. *Ba. vogeli* is generally the least severe, and *Ba. canis* is intermediately pathogenic in dogs [172]. Most common acute phase signs include fever, lethargy, thrombocytopenia, and varying degrees of hallmark hemolytic anemia, followed by chronic infection if untreated, which may be subclinical [173]. Severity of anemia is not necessarily mirrored by level of parasitemia and thus suggests that host factors play a role in inducing anemia in addition to direct erythrolysis caused by *Babesia* [172,173].

#### 5.1.3. Hematologic and Immunological Effects

The presence of a *Babesia* co-infection in dogs with CanL may complicate treatment decisions, as anemia can be seen during both infections. However, the types of anemia induced by each parasite can be distinct. To differentiate, a Coombs’ or agglutination test can be performed, which is sometimes positive in dogs with hemolytic anemia due to babesiosis but should be negative if nonregenerative anemia due to *L. infantum* is present [174,175]. Perhaps due to the different mechanisms driving anemia, synergistic anemia during co-infection has not been described but may be more apparent in young dogs or acutely after infection, as adult dogs seem to be predominately subclinical in the chronic phase of babesiosis.

Despite both parasites being protozoa, these two families are quite divergent, and there is no cross-reactivity between serological tests for canine *Babesia* or *Leishmania* exposure [176,177].

### 5.2. Trypanosoma cruzi

#### 5.2.1. Microbe and Epidemiology

*Trypanosoma cruzi* are obligate intracellular protozoan kinetoplastid parasites transmitted either through feces of infected triatomine bugs or transmitted congenitally between mammalian hosts [30]. *T. cruzi* is the etiologic agent for American trypanosomiasis, or Chagas’ disease [30]. *T. cruzi* is endemic in the Americas, from the Southern U.S. throughout Central and South America, and commonly transmitted by *Triatoma gerstaeckeri*, *T. sanguisuga*, *T. dimidiata* and *T. infestans* [30,31,32,33,34,35,36,37,38,39].

#### 5.2.2. Diagnostic Challenges and Immunologic Effects

*Leishmania* species and *T. cruzi* are phylogenetically similar, and there is a significant degree of cross-reactivity between the genera on microscopic examination and serological tests [178,179,180]. Dependent on the antigen(s) used, IFAT may have lower cross-reactivity compared to ELISA [179]. Cross-reactivity complicates the ability to identify if a dog is actively experiencing a *Leishmania* co-infection with *T. cruzi*, previous exposure to either pathogen, or cross-reaction on a diagnostic test.

Using PCR, enhanced specificity has been shown [181,182]. However, if the parasite load of either organism is low in a relevant diagnostic sample such as whole blood, PCR may not be sensitive enough for detection depending on the target sequence used [181,183]. In the Mediterranean, it is possible for *L. infantum* and *Trypanosoma* spp. to be found in phlebotomine sand flies and in canine hosts, which could present diagnostic challenges [184]. Studies utilizing PCR to identify co-infections between *Leishmania* and *T. cruzi* are limited. In 2003, Bastrenta et al. (2003) screened human blood or cutaneous ulcer biopsies from Bolivia and found 21 of 29 patients (72%) amplified both *Leishmania* spp. and *T. cruzi* DNA [185]. Only one of these instances was identified by isoenzyme profile as *L. infantum*—all other instances were cutaneous leishmaniasis species [185]. In 2007, Mendes et al. (2007) screened 1100 cases of human blood from Amazonians, and 11 cases (1%) had amplified both *Leishmania braziliensis* and *T. cruzi* DNA, and seven cases (0.6%) had amplified *L. infantum* and *T. cruzi* DNA [186]. One study used PCR to screen dog blood samples from Venezuela and found 18/283 (6.4%) samples amplified *Leishmania* and *T. cruzi* DNA [187]. Canine co-infection with *Leishmania* and *T. cruzi* is possible. All three studies used gel-based, non-quantitative methods of PCR to determine amplification, and only Mendes et al. (2007) demonstrated that primer sets did not cross-amplify purified control parasite DNA [186]. Co-infection may be more common with cutaneous *Leishmania* species than *L. infantum*, which causes CanL.

Radioimmunoprecipitation assay (RIPA) distinguishes presence of *T. cruzi* antibodies from *L. infantum*, as it does not produce false positives for *L. infantum* [188]. Duprey et al. (2006) used RIPA to determine *T. cruzi* presence among samples with titers greater than 128 via indirect immunofluorescent assay (IIF) [188]. Of these RIPA-tested samples, 86/413 (21%) were positive for *T. cruzi* [188]. Meyers et al. (2021) used rapid tests on 100 canine samples to distinguish *T. cruzi* or *L. infantum* infections from cross-reactions on indirect fluorescent antibody (IFA) tests [189]. After accounting for three cross-reactive samples, the authors concluded a 2% seroprevalence for *L. infantum* [189]. In all, a combination of serological tests or PCR methods may be needed to determine co-infection of *T. cruzi* and *L. infantum* or a single infection.

There may be an immunological basis as to why co-infections between *Leishmania* and *T. cruzi* are not more commonly observed despite their overlapping endemicity. After an initial acute phase of *T. cruzi* infection, like *L. infantum*, a prolonged systemic subclinical infection with low to absent parasitemia can occur in dogs. This indeterminate stage can occur for the lifetime of the dog if untreated [31]. The immune mechanisms that lead to control of *T. cruzi* infection are very similar to those offering protection from *L. infantum* infection.

Replication of both intracellular parasites is controlled by a Th1 immune response [190]. IFN-γ and IL-2 production by Th1 cells in response to *T. cruzi* infection increases parasite uptake by macrophages, induces humoral responses, and activates CD8^+^ T cells all contributing to parasite control [191]. As previously discussed, Th1 immunity limits *L. infantum* replication and survival [56,57]. Considering humoral immunity, antibodies specific for *T. cruzi* surface glycoproteins interact with extracellular parasites and complement to induce parasite lysis [192]. Anti-Galα1,3-Galβ1,4-GlcNAc (α-Gal) antibodies are induced to high levels by both *Leishmania* and *T. cruzi*, and both parasites express this glycoprotein; therefore, cross-reactive antibodies produced by one parasitic infection may limit nascent infection by the other species before it is able to establish [193].

Together, we hypothesize that the overlap in protective adaptive immune mechanisms shared against *Leishmania* and *T. cruzi* infections is sufficient to limit concurrent infection by both species in the same animal. Still, a few cases of dogs PCR-positive for both pathogens have been reported [185,186,187]. Both parasitic infections are capable of inducing T cell exhaustion during the chronic phase (Figure 1C); therefore, immunosuppression due to either advanced CanL or Chagas disease may allow a co-infection to occur in some cases [191,194]. More surveillance using molecular PCR methods and parasite cultivation would be needed to solidify the degree of natural co-infection occurring in dogs between these two related pathogens.

### 5.3. Toxoplasma gondii

#### 5.3.1. Microbe and Epidemiology

*Toxoplasma gondii* are obligate intracellular apicomplexan parasites. *T. gondii* is globally distributed, and there is a high burden of infection in mammals worldwide, thus dogs in *Leishmania*-endemic areas are exposed [40,41]. A Brazilian study of 66 *L. infantum*-seropositive dogs found 59% were co-seropositive for *T. gondii* [130]. In another study from Brazil, 8 out of 14 *L. infantum*-infected dogs were co-seropositive for *T. gondii* [195]. No association between the titer of anti-*Leishmania* or anti-*Toxoplasma* antibodies was observed [41]. In another study, there was significant skew of *T. gondii* seropositivity in dogs that were also seropositive for *L. infantum* by Chi-squared test, which suggests that there is a *T. gondii* predisposition in *L. infantum*-exposed dogs [196]. In an *L. infantum*-endemic area of Spain, 58.7% of 46 dogs were seropositive for *T. gondii* exposure; however, no significant association was found [197]. Due to large species differences between apicoplastids and kinetoplastids, anti-*Toxoplasma* antibodies are not thought to cross react with anti-*Leishmania* antibodies [178].

#### 5.3.2. Immunological Effects

Dogs are intermediate *T. gondii* hosts infected by ingesting oocysts shed by the definitive host, cats, or through predation of infected hosts [198]. *T. gondii* parasites then invade the intestinal epithelium and disseminate [198]. After the dissemination phase, tachyzoites convert into bradyzoites and form cysts to evade immune responses that can remain latent for years [198]. Cell-mediated immunity maintains bradyzoites in latent form and the infection is usually associated with a low degree of morbidity and mortality in dogs [190,198]. However, in rare cases, cutaneous or systemic toxoplasmosis has been documented in dogs receiving immunosuppressive treatments for other conditions [78,199,200]. Several of these cases were fatal, highlighting the potential severity of toxoplasmosis in dogs if reactivated. CanL induces CD4^+^ T cell exhaustion in severe chronic stages, associated with systemic expression of T cell inhibitory receptors, ligands, and regulatory cytokines [76]. These pathways can result in off-target suppression of bystander T cells [76]. This raises the possibility of *T. gondii* reactivation during late-stage CanL. Cutaneous lesions are a well-documented clinical sign of CanL, and cutaneous lesions due to *T. gondii* arising in immune-exhausted dogs may be attributed to CanL, and reactivation may go unrecognized if immunohistochemical staining is not performed. Due to the lethality observed in immunosuppressed dogs undergoing reactivation, it is likely that reactivation of *T. gondii* in a dog with CanL would also result in death. However, while a productive Th1 immune response is maintained in dogs with CanL, latent *T. gondii* infection seems to cause negligible exacerbation [201].

## 6. Helminthic Co-Infections

### 6.1. Helminthes

A variety of intestinal helminths infect dogs in *L. infantum*-endemic areas [202,203]. In a cohort of 93 dogs from Brazil, *Ancylostoma caninum, Toxocara canis, Ancylostoma braziliense, Trichuris vulpis* and *Dipylidium caninum* were investigated in relation to *L. infantum* serology [204]. No significant differences in amount of adult worm recovery were observed between *L. infantum*-seropositive or seronegative dogs, but the presence of the gastrointestinal cestode *Dipylidium caninum* was significantly correlated with *L. infantum* seroreactivity [204]. Guardone et al. (2013) found no statistical association between helminth infection and *L. infantum* serology among 265 dogs in Italy [205]. However, *Dipylidium caninum* was not assessed. In humans with visceral leishmaniasis due to *L. donovani,* no link was found between intestinal helminths and VL disease severity [206].

### 6.2. Dirofilaria immitis

#### 6.2.1. Epidemiology and Clinical Disease

*D. immitis* is a microfilarial worm that causes heartworm disease in dogs. It is spread by mosquito vectors worldwide and is endemic in areas with CanL, such as the Mediterranean basin, Brazil, and the U.S. [42,44,45,46,47,85,207,208]. *D. immitis* infects cardiopulmonary tissue, eliciting tissue damage including cardiomegaly, pulmonary artery enlargement, and congestive heart failure [209]. Dogs can be subclinical, but as microfilaria burden rises, weight loss, fatigue, exercise intolerance, and persistent cough can be seen in combination with progressive, regenerative anemia and hemoglobinuria due to intravascular hemolysis [209].

A study of 118 dogs from Spain showed 29 microfilaria-infected dogs had significantly increased severity of clinical signs when co-infected with *L. infantum* [42]; however, dogs infected with *L. infantum* did not have more severe signs of CanL if they were also positive for microfilaria. This study also observed a lower prevalence of *Wolbachia* in microfilaremic dogs co-infected with *L. infantum* [42]. In southern Portugal, 8.3% of 230 dogs were co-infected with *L. infantum* and *D. immitis* [43]. Additional studies found no association between *D. immitis* and clinical CanL in dogs [20,130].

#### 6.2.2. Immunological Effects

Heartworm infection in dogs is associated with a mixed Th1/Th2 response and peripheral eosinophilia [44,210]. As discussed above, eosinophils may contribute to protection against *Leishmania* infection [211]. *D. immitis* infection in dogs is complicated by the presence of endosymbiotic *Wolbachia* bacteria within worms [210]. *D. immitis* is thought to induce a Th2 response, while *Wolbachia* is thought to be targeted by Type 1 immunity [210]. IL-4 and IL-10 mRNA are significantly higher in whole blood from microfilaremic versus amicrofilaremic dogs [47].

Due to the lack of studies finding a statistical association between *D. immitis* and *L. infantum* exposure or clinical synergy, we hypothesize that subclinical *D. immitis* in dogs has little effect on CanL immune responses. Supporting this rationale, use of macrocyclic lactones to prevent microfilariae had no significant effect on the likelihood of *L. infantum* seropositivity in dogs from Portugal [43].

## 7. Fungal Co-Infection

### 7.1. Paracoccidioides brasiliensis

#### 7.1.1. Epidemiology and Clinical Disease

*Paracoccidioides brasiliensis* is a fungus that causes the systemic infection paracoccidioidomycosis. *P. brasiliensis* is endemic in Central and South America with the majority of human and canine cases occurring South America [212]. Dogs in Brazil are highly exposed with seroprevalence as high as 89.5% in rural areas [213]. Despite a large burden of disease in humans, dogs infected with *P. brasiliensis* are largely resistant to disease [214]. Case reports of disease in dogs describe marked lymphadenomegaly, apathy, loss of appetite, poor condition, emaciation, hepatosplenomegaly, and dermatitis [48,215,216]. Lymph node biopsies showed granulomatous lymphadenitis with numerous fungal yeast forms and clinicopathology showed neutrophilia, nonregenerative anemia, and thrombocytopenia [48,215,216].

In a cohort of 200 dogs from Brazil, *P. brasiliensis* seropositivity was significantly associated with also being seropositive for *Leishmania* (OR = 25.73) [217]. This group also observed a higher percentage (67.8%) of *P. brasiliensis*-seropositive dogs among *Leishmania*-seropositive dogs in a different region of Brazil [218]. The authors do not believe antibody cross-reactivity was occurring because there was not a significant association between the raw absorbance values against *Leishmania* antigen and *P. brasiliensis* gp63 antigen [217]. Therefore, dogs with CanL may have increased susceptibility to *P. brasiliensis*.

#### 7.1.2. Immunological Effects

Monocytes and macrophages are the main cell types responsible for killing of *P. brasiliensis* [219]. Soares et al. hypothesize that prostaglandins reduce the ability of monocytes to kill *P. brasiliensis* because treatment with a cyclo-oxygenase inhibitor significantly increased monocyte fungicidal activity [220]. *L. infantum*-derived lipophosphoglycan extract has been shown to induce COX2 expression and prostaglandin E2 production by macrophages [221].

Macrophage *P. brasiliensis* fungicidal activity is enhanced by IFN-γ and TNF-α, and a Th1 response is associated with protection [222]. The immunoregulatory cytokine IL-10 antagonizes IFN-γ activity and is associated with susceptibility to *P. brasiliensis* [222]. Patients were significantly more likely (OR = 5.8) to have a single nucleotide polymorphism in the IL-10 gene, resulting in enhanced IL-10 expression [223]. Increased secretion of IL-10 and transforming growth factor beta (TGF-β) was measured from patient monocytes compared to healthy control monocytes [224]. *P. brasiliensis*-susceptible mice show increased dendritic cell IL-10 and increased CTLA4 protein expression by T regulatory cells in humans with active disease [225,226]. CTLA4 is an inhibitory receptor expressed by T cells that contributes to T cell exhaustion [227]. As CanL advances, IL-10 is produced systemically [76,228]. Thus, regulatory pathways induced by *P. brasiliensis* and *L. infantum* infection could synergistically act to deregulate Th1 cell function, expediting immune exhaustion and leading to fungal and parasite outgrowth in dogs with visceral leishmaniasis.

## 8. Effects on Diagnosis and Consideration of Cross-Reactions

In general, the pathogens discussed in this review can be detected by PCR methods or the associated antibodies detected by a serological method, and often a combination of diagnostic tests are utilized to understand the patient’s infection status. As an example for *Leishmania*, a study by da Costa Oliveira et al. (2021) found 89.4% of 66 Brazilian dogs identified by serology had a positive result for *Leishmania* spp. by either immunohistochemistry or culture [130]. Different specimens may also be used for diagnostic testing, such as lymph node or spleen aspirates, although the majority of testing uses blood or sera samples due to ease of blood collection compared to more invasive techniques [85]. One study detected *L. infantum* DNA in brain and spinal cord samples by qPCR despite lack of neurological signs in the dogs before euthanasia [195]. Evaluation of the dog’s clinical presentation and history in accordance with the diagnostic results is appropriate for forming a diagnosis and subsequent treatment options.

There are different sensitivity rates between serological tests and PCR tests. For example, de Sousa et al. (2013) found higher frequencies of canine samples being seropositive by ELISA and/or IFAT than PCR-positive for *Leishmania*, *Ehrlichia* spp., and *Babesia* spp. [171]. Furthermore, there can be transient PCR positivity [229,230]. PCR is often more difficult to detect the specific pathogen’s nucleic material, even during an active infection [8]. Consequently, the serological test may detect antibodies at a larger frequency, but the timing of exposure and/or infection cannot be confirmed solely with serological testing [8]. According to Otranto et al. (2009), diagnostic tests may not be sensitive enough to distinguish between healthy or subclinical dogs and chronically ill dogs [229].

A diagnostic challenge for *Leishmania* and some of these pathogens is the possibility of diagnostic cross-reaction, especially on serological tests. The most documented cross-reaction with *Leishmania* is *T. cruzi*, another kinetoplastid [179]. The detected species of each genus may vary between tests. A case study documented a dog in Brazil having a cross-reaction on *Leishmania* IFAT and *T. cruzi* IFAT, although PCR and sequencing confirmed the dog to be co-infected with *L. infantum* (*chagasi*) and *T. evansi* [231]. Additionally, it can be difficult to determine whether a seropositive result is from true presence of specific antibodies to two species or a cross-reaction. For example, da Silva Krawczak et al. (2015) found different seropositivity frequencies between IFAT, ELISA, DPP, and rK39 RDT (Kalazar Detect Canine Rapid Test) for *Leishmania*, *Ehrlichia*, and *Babesia* testing among urban pet dogs in Minas Gerais, Brazil; however, there were no cross-reactions between *Leishmania* and *Babesia* or *Ehrlichia* [176]. Similarly, de Sousa Oliveira et al. (2008) determined that there was no cross-reactivity between *Leishmania*, *Babesia*, and *Ehrlichia* by IFAT [177]. In another study, six dogs were seropositive for *Leishmania* and *Trypanosoma* but negative for these pathogens on PCR, and only 0.74% of PCR tests had positivity for both *Leishmania* and *Trypanosoma* [232]. No cross reactions were detected among 160 total canine sera samples used for mixed indirect IFAT for *Leishmania* and *Ehrlichia*, and the authors determined that mixed IFAT is specific for CanL and *Ehrlichia* [233].

In contrast, other studies have reported presence of cross-reactions. Zanette et al. (2014) demonstrated presence of cross-reactivity between *T. cruzi* and *Leishmania* on ELISA and IFAT [178]. Troncarelli et al. (2009) found cross-reactions between *Leishmania* spp. and *T. cruzi* on IFAT, as 16.5% of the 200 samples were positive for both antibodies, and the authors suggested that both PCR and direct parasitological examination is needed for CanL diagnosis [179]. Similarly, Attipa et al. (2019) suggest that dogs with clinical CanL be tested for *E. canis* co-infection by both PCR and serology [20].

## 9. Treatment Implications and Complexities

Although more research during natural infections is needed to assess therapeutic management strategies and prognosis of co-infections, there is evidence that co-infections affect the dog’s immunity against *L. infantum* and subsequent progression of disease. Dogs with CanL and co-infections (9 of 99 tested dogs in Portugal) with either *E. canis*, *B. canis,* and *Rickettsia conorii* had shorter survival time (*p* = 0.0142) [54]. Additionally, Toepp et al. (2019) found that dogs with multiple tick-borne co-infections had statistically significant increased risk for progressed CanL and increased risk for mortality [10].

The severity of clinical signs in a co-infected or co-exposed patient may be subjective and not have consistent record of scale (such as lack of tissue measurements or degree of skin lesions). As a result, there may be inadequate or underreporting of particular signs for various co-infections. According to da Costa Oliveira et al. (2021), clinical signs were not made worse by co-infections with *T. gondii*, *Ehrlichia* spp., or *Anaplasma* spp., although 91% of the 66 dogs had clinical CanL with the most common clinical signs being splenomegaly, onychogryphosis, and furfuraceous desquamation of skin [130]. Additionally, contribution of clinical signs to a specific pathogen is nearly impossible for a dog with these co-infections, especially since these infections often have similar physical exam findings and similar hematologic and serum chemistry findings.

Treatment for CanL is often a combination of allopurinol and an antimonial or miltefosine [234]. The bacterial diseases discussed in this review are largely treated with a course of doxycycline. Among five dogs co-infected with *L. infantum* and *E. canis*, treatments included meglumine antimoniate, allopurinol, and doxycycline [100]. De Tommasi et al. (2013) recommend treating co-infections simultaneously [100].

Barriers to successful chemotherapy include relapses, long courses of drug administration, toxicities, antimicrobial resistance, and cost [229]. Dogs with CanL are often infected for life, and progression of clinical signs may occur earlier in life if co-infections are also present [10,234]. Recrudescence is common among dogs with CanL [234]. Prolonged and/or lifelong therapy for dogs with CanL can be taxing on owners, and frequent veterinary visits are necessary for continued assessment. Bacterial or parasitic co-infections may also be chronic, with re-infections possible, thus adding complexity to the monitoring and therapeutic plan for a patient.

## 10. Prevention Strategies

Prevention for these infections is based on the respective vector and/or environmental setting. As *Leishmania* is transmitted by phlebotomine sand flies, endemic areas can employ strategies to ward off sand fly bites. Insecticides include deltamethrin-impregnated dog collars, topical permethrin-based products, and spray repellants [235]. For environmental control, mesh screens can be applied to windows or open areas where dogs are housed, breeding sites for sand flies can be eliminated, and dogs can be kept inside from dusk to dawn when sand flies are most active [235]. In non-endemic areas, testing of dogs and bitches before breeding can prevent vertical transmission, especially among dog breeds at most risk, and preventing dog fights with known *Leishmania*-infected dogs can limit horizontal transmission [235].

For control of the three main bacterial co-infections and *Babesia* spp., tick vectors can be targeted and prevented. A variety of tick preventive medications are commercially available, and may vary by country, and the effective spectrum of the preventive should correspond to the area’s tick species prevalence. Likewise, a heartworm preventive medication should be administered year-round in endemic areas. The concomitant use of broad-spectrum preventive products could protect dogs against vectored parasites. For example, Abbate et al. (2018) found that concomitant administration of topical fipronil/permethrin and oral afoxolaner/milbemycin oxime in dogs during a six-month period was efficacious in preventing main tick-borne bacterial infections, seroconversion of any *L. infantum* infection, and certain endoparasitic infections [236]. Environmental control can include mosquito abatement and removing ticks when observed on dogs.

For CanL management in China, treatment of *L. infantum*-infected dogs and control of vectors are instituted [237]. In Shanghai, China, dog owners have been given a sulfa drug by governmental authorities for *T. gondii* control since 2002 [237]. In Brazil, prevention methods for CanL include ectoparasiticides, vaccines, and dog culling, which has ethical controversy [229,238]. In general, surveillance for animal and human vector-borne diseases can improve public health [229]. Other strategies for prevention can include reducing free-roaming dogs and improved kennel management [229].

In all, the most effective prevention measures for these diseases are controlling exposure to the respective vector. While there are vaccines available in certain countries for *Leishmania*, *Borrelia*, and *Babesia*, the feasibility and effectiveness of these vaccines in preventing transmission have been limited [145,235]. Therefore, a combination, or multimodal approach, of prevention strategies is needed to decrease risk of these infections.

## 11. Concluding Remarks

Experimental models of visceral leishmaniasis have elucidated the pathogenesis of *L. infantum*. However, these oversimplified models cannot replicate the clinical picture occurring in outbred, naturally acquired CanL. Dogs throughout the world are regularly exposed to infectious organisms which may or may not cause disease. Therefore, rarely do dogs encounter *L. infantum* in a vacuum, and instead, dogs remain without clinical disease or develop CanL amidst an array of infectious exposures, which may modify the immunopathogenesis of CanL and offer a more accurate picture of the disease.

Herein, we have described eight common co-infections incurred by dogs in *L. infantum*-endemic areas and explored how these co-infections may synergize to impact CanL immune responses or clinical progression. This work is not comprehensive, as the full breadth of relevant CanL co-infections is not known, and we expect each co-infecting species will have a unique interplay with *Leishmania* immunity and CanL disease. We found pathogens capable of interfering with arms of the *Leishmania* immune response, such as macrophage microbicidal activity or Type 1 T cell polarization, were most likely to impact CanL disease progression. This implies the role of immune dysregulation is greater than pathology due to the co-infecting pathogen itself.

Evident in the literature was a theme of tick-borne pathogens being particularly common co-infections during CanL and generally inducing negative consequences in co-infected dogs. This is not overall surprising, as dogs are highly exposed to ticks, which can carry multiple types of pathogens. This highlights the need for use of tick and sand fly preventives for dogs in *Leishmania*-endemic areas, which are available and highly effective. Further research is needed on modulation of immunity in co-infected dogs during CanL in order to improve diagnostics, treatment decisions, and limit the spread of *L. infantum* among dogs. Importantly, as a One Health model, similar immune mechanisms may occur in VL patients encountering co-infections with human pathogens like lymphatic filariasis or HIV.

## Figures and Tables

**Table 1:** Main pathogens involved in co-infection with canine leishmaniosis.

Pathogen	Type of Pathogen	Main Vector(s)	Region(s) Primarily Found	Reference(s)
*Leishmania infantum*	Protozoa	*Phlebotomus* spp.	Mediterranean basin Southern Europe Northern Africa	[1,2]
		*Lutzomyia longipalpis*	South America	[2,3]
		None	North America (enzootic)	[6,7]
*Ehrlichia canis*	Bacteria	*Rhipicephalus sanguineus*	North America South America Mediterranean basin	[13,14,15,16]
*Ehrlichia ewingii*	Bacteria	*Amblyomma americanum*	North America	[13,14,15,17]
*Ehrlichia chaffeensis*	Bacteria	*Amblyomma americanum*	North America	[13,14,15]
*Anaplasma phagocytophilum*	Bacteria	*Ixodes scapularis*	North America	[18]
		*Ixodes pacificus*	Western U.S.	[18]
		*Ixodes ricinus*	Europe	[18,19]
*Anaplasma platys*	Bacteria	*Rhipicephalus sanguineus*	Brazil Europe	[9,16,20]
*Borrelia burgdorferi*	Bacteria	*Ixodes scapularis*	North America	[21,22,23]
		*Ixodes pacificus*	Western U.S.	[21]
*Borrelia garinii*	Bacteria	*Ixodes ricinus*	Europe	[19,22,24]
*Borrelia afzelii*	Bacteria	*Ixodes ricinus*	Europe	[22,24]
*Babesia canis*	Protozoa	*Dermacentor reticulatus*	Europe	[25,26,27]
		*Rhipicephalus sanguineus*	Brazil	[25,28]
*Babesia vogeli*	Protozoa	*Rhipicephalus sanguineus*	Brazil	[28]
*Babesia gibsoni*	Protozoa	*Haemaphysalis bispinosa*	Asia	[29]
		*Haemaphysalis longicornis*	Asia	[27]
*Trypanosoma cruzi*	Protozoa	*Triatoma gerstaeckeri*, *T. sanguisuga*	North America	[30,31,32,33,34,35,36]
		*T. dimidiata*	Central America	[33,37]
		*T. infestans*	South America	[33,38,39]
*Toxoplasma gondii*	Protozoa	None	South America North America Europe Asia	[40,41]
*Dirofilaria immitis*	Helminth	*Aedes, Anopheles, Culex*	North America, South America, Europe	[42,43,44,45,46,47]
*Paracoccidiodes* *brasiliensis*	Fungi	None	South America Central America	[48]

## Data Availability

Not applicable.
